# (Anti)-mattering and its association with child maltreatment and loneliness: evidence from the German versions of the general mattering and anti-mattering scales

**DOI:** 10.1007/s00406-026-02327-6

**Published:** 2026-07-15

**Authors:** Matthias A. Reinhard, Djuna Schwedler, Maxime Wendlinger, Alina Zeller, Franziska Appl, Julia I. Kunz, Johannes Wolf, Andrea Jobst, Frank Padberg, Gordon L. Flett, Stephan Goerigk

**Affiliations:** 1 Department of Psychiatry and Psychotherapy, University Hospital, LMU Medizin, Ludwig-Maximilians-University, Nussbaumstrasse 7, 80336 Munich, Germany; 2 DZPG (German Center for Mental Health), Partner Site Munich/Augsburg, Munich, Germany; 3 Charlotte Fresenius Hochschule, Infanteriestrasse 11A, 80797 Munich, Germany; 4 York University, Toronto, Canada; 5 Department of Psychological Methodology and Assessment, Ludwig-Maximilians-University, Leopoldstrasse 13, 80802 Munich, Germany

**Keywords:** Mattering, Anti-mattering, Loneliness, Adverse childhood experiences, Psychological well-being, Psychometric validation

## Abstract

**Background:**

Mattering and anti-mattering are closely associated with mental health outcomes, including loneliness. Mattering describes the perceived sense of being important to others, whereas anti-mattering reflects feelings of being ignored, unvalued, or insignificant. Both constructs may be shaped by adverse childhood experiences, including child maltreatment. Therefore, the present study examined the relationships among mattering, anti-mattering, child maltreatment, and loneliness, while also evaluating the psychometric properties of the German versions of the General Mattering Scale (GMS) and the Anti-Mattering Scale (AMS).

**Methods:**

In a German-speaking online sample (*N* = 284), we assessed child maltreatment, mattering, anti-mattering, loneliness, psychological distress, quality of life, and social network size. Mediation analyses examined whether mattering and anti-mattering mediated the association between child maltreatment and loneliness. Internal consistency, factor structure, and associations with related constructs were examined to evaluate the German GMS and AMS.

**Results:**

Child maltreatment was associated with lower mattering, higher anti-mattering, and greater loneliness. Both mattering and anti-mattering partially mediated the association between child maltreatment and loneliness, with anti-mattering showing the stronger indirect association. The German GMS and AMS demonstrated good internal consistency, and a two-factor model supported mattering and anti-mattering as distinct but related constructs. Their associations with loneliness, psychological distress, quality of life, social network size, and child maltreatment supported criterion validity.

**Conclusions:**

The German GMS and AMS are reliable and valid instruments. Findings highlight the relevance of mattering and especially anti-mattering for understanding loneliness and its links to early adversity, suggesting their potential importance as clinical and psychotherapeutic targets.

**Clinical trial registration:**

The validation process was preregistered separately for the GMS and AMS on the Open Science Framework (OSF): https://osf.io/8tsey/?view_only=cb9cb335a19b46faa7e9e00a5e21668b; https://osf.io/yvmpa/?view_only=1eb275b272a44fe88bb43e87b6e12163.

## Introduction

Loneliness is increasingly recognized as a major public health concern associated with impaired psychological well-being, and increased risk for mental and physical health problems. Defined as the perceived gap between desired and actual social relationships [1], loneliness can be conceptualized as a subjective and relational experience. Importantly, individuals may experience profound loneliness even in the presence of a large social network when they perceive themselves as unseen, unimportant, or emotionally disconnected from others [2, 3]. Accordingly, interpersonal perceptions concerning one’s relational significance and social value may represent important psychological correlates of loneliness and emotional distress [2, 3].

One construct that may be particularly relevant in this context is *mattering*. Mattering is a core concept within positive psychology [4], and refers to the experience of feeling seen, heard, and valued, as well as having meaningful social connections where one’s presence is noticed and appreciated [4–6]. Individuals with a strong sense of mattering perceive themselves as noticed, cared for, and emotionally relevant within their interpersonal relationships and broader social environment. Previous research has linked mattering to numerous indicators of psychological well-being, including lower depressive symptoms [7, 8], and lower loneliness [3]. These findings suggest that mattering may function as an important protective interpersonal resource that contributes to positive well-being [9].

More recently, researchers have emphasized the importance of distinguishing mattering from anti-mattering [10]. *Anti-mattering* refers to experiences of perceived insignificance, invisibility, neglect, and the sense that one does not matter to others. Although mattering and anti-mattering are negatively associated, anti-mattering is increasingly conceptualized not simply as low mattering, but as a qualitatively distinct interpersonal experience involving perceived relational devaluation and interpersonal irrelevance [10]. Empirical findings suggest that anti-mattering demonstrates unique associations with psychological distress beyond low mattering and may be more strongly associated with psychological vulnerabilities such as depression, loneliness, social anxiety, and low self-esteem, as well as maladaptive behaviors like self-harm and poor self-care [3, 10–14]. Indeed, a recent meta-analysis [8] found a stronger link between depression and anti-mattering than with mattering. In addition, the robust association of loneliness with mattering and anti-mattering suggests that loneliness is a deeply relational and perceptual experience rooted in feeling unnoticed, undervalued, or expendable [3], and not merely a function of limited social contact. Therefore, examining both subjective relational experiences (i.e., loneliness) and objective indicators of social connectedness (i.e., social network size) in the context of mattering and anti-mattering may provide a more comprehensive understanding.

The distinction between mattering and anti-mattering may be especially relevant in the context of adverse relational experiences during childhood. Child maltreatment, including emotional abuse, emotional neglect, physical abuse, and sexual abuse, has consistently been associated with elevated loneliness and interpersonal difficulties later in life [15, 16]. Developmental and attachment-oriented perspectives propose that early adverse interpersonal experiences may shape internalized relational schemas concerning self-worth, belongingness, and interpersonal significance [17]. Such experiences may communicate powerful relational messages that one is unimportant, invisible, unwanted, or undeserving of care, and may subsequently contribute to chronic expectations of interpersonal insignificance and relational disconnection.

From this perspective, anti-mattering may be relevant to understanding the association between child maltreatment and loneliness. Individuals exposed to child maltreatment may report stronger feelings of insignificance and invisibility that persist into adulthood. Consequently, loneliness may be associated not only with reduced social contact, but also with perceptions of not mattering to others. In contrast, mattering may function as a protective relational resource that is associated with lower feelings of disconnection and emotional isolation.

The General Mattering Scale (GMS) and the Anti-Mattering Scale (AMS) represent two brief and widely used self-report instruments designed to assess perceived mattering and anti-mattering [10, 18]. Developed to operationalize Rosenberg’s theory of mattering, the GMS consists of five self-report items designed to assess individuals’ general sense of interpersonal significance and demonstrated reliability and construct validity. Building on this framework, Flett and colleagues [10] developed and established the AMS as a five-item unidimensional measure. Existing studies have demonstrated satisfactory psychometric properties of both instruments in English-speaking and several international samples [10–14]. However, reliable and valid German-language versions of these instruments are currently lacking. Cross-cultural validation is particularly important because the meaning and expression of relational constructs may vary across linguistic and cultural contexts. Furthermore, establishing reliable and valid German-language versions of the GMS and AMS is a prerequisite for future research and clinical applications in German-speaking contexts.

Against this background, the present study pursued two aims. The *primary aim* was to investigate whether mattering and anti-mattering are associated with the relationship between child maltreatment and loneliness. Specifically, we examined whether mattering and anti-mattering mediate the association between recalled child maltreatment and loneliness in adulthood. Based on previous theoretical and empirical work, we expected child maltreatment to be associated with lower mattering, higher anti-mattering, and greater loneliness. Furthermore, we expected anti-mattering to demonstrate stronger associations with loneliness than mattering. The *secondary aim* of the study was to evaluate the psychometric properties of the German versions of the GMS and AMS. Specifically, we examined internal consistency, factorial validity, and associations with related psychological and interpersonal variables in order to assess the suitability of the scales for use in German-speaking populations.

## Materials and methods

### Study design

The psychometric properties of the German versions of the GMS and the AMS were evaluated using a cross-sectional survey design. The validation process was preregistered separately for the GMS and AMS on the Open Science Framework (OSF): https://osf.io/8tsey/?view_only=cb9cb335a19b46faa7e9e00a5e21668b; https://osf.io/yvmpa/?view_only=1eb275b272a44fe88bb43e87b6e12163.

All study procedures were conducted in accordance with the ethical standards of the Declaration of Helsinki and its later amendments. Ethical approval was obtained from the Ethics Committee of Charlotte Fresenius University of Applied Sciences, Munich (project no.: 2024-006SG). Informed consent was obtained electronically from all participants prior to participation.

### Sample

The final sample comprised 284 participants, of whom 201 (70.8%) identified as women, 81 (28.5%) as men, and 2 (0.7%) as non-binary. The mean age was 33.67 years (SD = 15.20), and the average duration of formal education was 16.20 years (SD = 3.53). A total of 40.8% of the sample reported having prior experience with psychiatric or psychotherapeutic treatment. Regarding relationship status, the majority of participants (61.3%) reported being in a committed relationship or married. Additionally, 35.2% identified as single, while smaller proportions reported being divorced (1.1%), widowed (1.4%), or separated (1.1%).

### Procedure

The GMS and AMS were translated from English into German by M.A.R. and S.G. Following this, the German versions were independently back-translated into English by a native English speaker to ensure linguistic and conceptual equivalence between the original and translated versions (see Table 1). Unlike the original English versions, only the endpoints of the German response scales were verbally labeled (“überhaupt nicht” and “sehr”), whereas the intermediate response categories remained unlabeled due to difficulties in finding sufficiently precise German equivalents. Importantly, participants nevertheless had access to the full four-point response scale.

**Table 1:** Loadings for the General Mattering Scale and Anti-Mattering Scale from the correlated two-factor model

Scale	Items	German translation	Factor loadings	z value	*P* value
GMS	How important are you to others?	Wie wichtig sind Sie für andere?	0.79		
GMS	How much do others pay attention to you?	Wie viel Aufmerksamkeit erhalten Sie von anderen?	0.62	9.44	< 0.001
GMS	How much would you be missed if you went away?	Wie sehr würden Sie vermisst werden, wenn Sie weggingen?	0.71	10.51	< 0.001
GMS	How interested are others in what you have to say?	Wie interessiert sind andere daran, was Sie zu sagen haben?	0.56	8.47	< 0.001
GMS	How much do other people depend upon you?	Wie sehr sind andere Menschen auf Sie angewiesen?	0.46	7.03	< 0.001
AMS	How much do you feel like you don’t matter?	Wie sehr fühlen Sie, dass Sie nicht von Bedeutung sind?	0.59		
AMS	How often have you been treated in a way that makes you feel like you are insignificant?	Wie oft sind Sie auf eine Weise behandelt worden, die Ihnen das Gefühl vermittelt, dass Sie unbedeutend sind?	0.79	9.12	< 0.001
AMS	To what extent have you been made to feel like you are invisible?	In welchem Ausmaß ist Ihnen das Gefühl vermittelt worden, als ob Sie unsichtbar sind?	0.80	9.56	< 0.001
AMS	How much do you feel like you will never matter to certain people?	Wie sehr fühlen Sie, dass Sie für bestimmte Leute niemals von Bedeutung sein werden?	0.62	8.19	< 0.001
AMS	How often have you been made to feel by someone that they don’t care what you think or what you have to say?	Wie oft wurde Ihnen von jemandem das Gefühl vermittelt, dass es ihm/ihr egal ist, was Sie denken oder was Sie zu sagen haben?	0.71	8.94	< 0.001

Items are reproduced for scholarly purposes. No commercial use is intended

*GMS* general mattering scale, *AMS* anti-mattering scale

Data was then collected via a fully anonymous online survey. Participants were recruited through social media platforms and printed flyers distributed in public locations such as universities and libraries. The survey began with the assessment of sociodemographic variables, including gender, age, relationship status, educational attainment, and current or past experience with psychiatric or psychotherapeutic treatment. Following this, participants completed seven self-report questionnaires.

Inclusion criteria were: (a) minimum age of 18 years, and (b) sufficient proficiency in the German language to comprehend study materials and questionnaire content. While the sample was recruited predominantly non-clinically, individuals with prior clinical experience were not excluded. In accordance with established recommendations for psychometric research [19, 20], a minimum sample size of *N* = 238 was preregistered to ensure adequate statistical power for the planned analyses.

### Measures

*General Mattering Scale*: The GMS [18] consists of five items measuring the extent to which individuals perceive themselves as significant to others. Items are rated on a 4-point Likert scale from 1 (“Not at all”) to 4 (“A lot”), and summed to yield a total score (range: 5–20), with higher scores reflecting stronger feelings of mattering. The GMS has shown good internal consistency in previous research (e.g., α = 0.85 [7]).

*Anti-Mattering Scale*: The AMS [10] is a five-item self-report instrument assessing the perception of being insignificant or unimportant to others. Items are rated on a 4-point Likert scale ranging from 1 (”Not at all”) to 4 (“A lot”), with higher scores indicating greater levels of perceived anti-mattering. Total scores are obtained by summing item responses (range: 5–20). The AMS has demonstrated good internal consistency (α = 0.86 [10]).

*Loneliness*: The UCLA Loneliness Scale (UCLA-LS; [21]) comprises 20 items assessing subjective experiences of loneliness across three dimensions: feelings of loneliness, perceived emotional isolation, and perceived social isolation. Responses are given on a 5-point scale ranging from 1 (“Not at all”) to 5 (“Totally”), with higher scores indicating greater loneliness. The scale has demonstrated strong psychometric properties, including split-half reliability of r_tt_ = 0.85 and internal consistency of α = 0.89 [21] (in the present sample: α = 0.92).

*Mental Health Symptoms*: The Depression Anxiety Stress Scale (DASS-21; [22]) is a 21-item self-report instrument measuring symptoms of depression, anxiety, and stress. Each of the three subscales contains seven items rated on a 4-point Likert scale ranging from 0 (“Did not apply to me at all”) to 3 (“Applied to me very much or most of the time”). Higher scores reflect greater symptom severity. The DASS-21 demonstrated good internal consistency in the present sample: ω = 0.88 (Depression), ω = 0.83 (Anxiety), ω = 0.85 (Stress), and ω = 0.89 for the total score.

*Quality of Life*: The WHO Quality of Life–BREF (WHOQOL-BREF; [23]) is a 26-item abbreviated version of the WHOQOL-100 assessing quality of life across four domains: physical health, psychological well-being, social relationships, and environmental context. It also includes two items on general quality of life and overall health. The overall score’s internal consistency in the current sample was α = 0.89.

*Social Network Size*: The Social Network Index [24] is a 12-item measure assessing the number of individuals’ social relationships across various domains (e.g., family, friends, coworkers). Participants report the number of people they interact with at least biweekly in each relationship category. Most items are rated on a scale from 0 to “7 or more”, while items concerning parents and romantic partners have specific categorical responses. In accordance with the published scoring recommendations for the Social Network Index, responses to Item 12 (group membership) were limited to a maximum of seven group members to prevent artificial inflation of network size estimates. A total score is calculated by summing up the number of social relationships for the 12 domains resulting in a range from 0 to 68. The internal consistency for the social network size in this sample was rather low (α = 0.40). However, as network domains (e.g., marital status, employment, religious participation) represent independent social roles rather than a single latent psychological trait, high internal consistency is not methodologically required or expected.

*Child Maltreatment*: The Childhood Trauma Questionnaire (CTQ; [25]) is a 28-item retrospective self-report instrument assessing five types of child maltreatment: emotional, physical, and sexual abuse, as well as emotional and physical neglect. Items are rated on a 5-point Likert scale ranging from 1 (“Never true”) to 5 (“Very often true”). Subscale scores are aggregated to yield a total index of childhood trauma. Internal consistency for the total score in the present sample was α = 0.89.

### Statistical analyses

All statistical analyses were performed using R version 4.5.0 [26]. The threshold for statistical significance was set at α = 0.05. For sets of multiple correlation analyses, p-values were corrected for multiple testing using the false discovery rate (FDR) procedure. Assumptions underlying parametric tests, including normality of residuals and homoscedasticity, were evaluated. Spearman correlations and robust estimation methods were used where appropriate to account for non-normal distributions. No missing data were detected in the dataset.

*Reliability analysis*: The internal consistency of the GMS and AMS were evaluated using McDonald’s omega. In accordance with the preregistered analysis plan, either McDonald’s omega or Cronbach’s alpha was to be reported depending on the underlying measurement model. Given the theoretical assumption of unidimensionality [10] and the appropriateness of a tau-congeneric model, McDonald’s omega was considered the preferred reliability estimate. To provide a more robust estimate of internal consistency, maximal split-half reliability (λ_4_) was calculated following the procedure outlined by Callender and Osburn [27, 28]. Traditional split-half reliability methods that rely on random item splits and Spearman-Brown corrections may yield biased estimates when tau-equivalence is violated [29]. Therefore, all possible item split combinations of the GMS or the AMS were examined within the full sample. For each split, the correlation between the two halves was corrected using the Spearman-Brown prophecy formula, with the split producing the highest reliability coefficient identified as the maximal lambda 4 (λ_4_) [30]. To mitigate potential sampling error and reduce the risk of overestimating reliability [29], a cross-validation strategy was employed, as recommended [30]. The total sample was randomly divided into two halves, with one half serving as the training set and the other as the test set. The maximal split identified in the training set was reapplied to the test set, and the resulting reliability coefficient was bias-corrected to yield a cross-validated maximal split-half reliability coefficient, denoted Max(_λ4_) [30]. This approach provides a more generalizable and stable estimate of internal consistency for the present sample [29].

*Confirmatory Factor Analysis*: To examine the factor structure of the GMS and AMS, a confirmatory factor analysis was conducted using maximum likelihood estimation based on a measurement-based model framework. To provide evidence for the distinctiveness of mattering and anti-mattering, we compared a single-factor model, in which all GMS and AMS items loaded onto a common latent factor, with a correlated two-factor model in which items loaded onto their respective constructs [31]. Furthermore, a higher-order model and a bifactor model were fit but consistently yielded non-convergence and negative latent variances regardless of identification constraints applied. Nested models were compared using a χ² likelihood-ratio-test, and global model fit was evaluated using multiple fit indices and interpreted according to established cut-off criteria [32, 33]. The indices included the chi-square test (²), root mean square error of approximation (RMSEA) with its 90% confidence interval and p-close statistic, standardized root mean square residual (SRMR), comparative fit index (CFI), and Tucker-Lewis index (TLI). Local model fit was further assessed using critical ratios, and modification indices in line with recommendations [34, 35]. Modification indices greater than 10 were considered suggestive of potential model misspecification [34].

*Measurement invariance*: Measurement invariance was examined using multi-group CFA in order to analyze whether the GMS and AMS measure the same underlying constructs equivalently across gender and across with vs. without prior treatment. First, a configural invariance model was estimated, allowing all parameters to vary freely across groups while maintaining the same factor structure. Next, metric invariance was tested by constraining factor loadings to equality across groups. Finally, scalar invariance was evaluated by additionally constraining item intercepts to equality. Model comparisons were conducted using χ² likelihood-ratio-tests.

*Mediation analysis:* A parallel multiple mediation analysis (with 10,000 bootstrap samples to estimate confidence intervals) was conducted using PROCESS Model 4 [36] to examine whether the relationship between child maltreatment and loneliness was mediated by the GMS and AMS.

## Results

### Mattering and anti-mattering

In the present sample, the mean score on the GMS was 14.89 (*SD* = 2.49) and the mean score on the AMS was 10.27 (*SD* = 3.22). Descriptive statistics for all study variables are presented in Table 2.

**Table 2:** Descriptive statistics and bivariate correlations of study variables

Variable	M (SD)	ρ with GMS	*P* value	ρ with AMS	*P* value	z value	*P* value
Mattering (GMS)	14.89 (2.49)	–		− 0.37	< 0.001***		
Anti-Mattering (AMS)	10.27 (3.22)	− 0.37	< 0.001***	–			
Loneliness (UCLA-LS)	1.84 (0.59)	− 0.47	< 0.001***	0.50	< 0.001***	0.54	0.59
Depressive symptoms (DASS-21)	8.70 (8.83)	− 0.27	< 0.001***	0.45	< 0.001***	2.98	0.003**
Anxiety symptoms (DASS-21)	6.25 (7.24)	− 0.24	< 0.001***	0.37	< 0.001***	2.08	0.04*
Stress symptoms (DASS-21)	11.14 (8.13)	− 0.17	0.003**	0.34	< 0.001***	2.67	0.01*
Quality of life (WHOQOL-BREF)	73.40 (11.96)	0.38	< 0.001***	− 0.49	< 0.001***	1.90	0.06
Social network size (SNI)	14.49 (7.56)	0.38	< 0.001***	− 0.20	< 0.001***	2.71	0.007**
Childhood trauma (CTQ)	36.76 (12.15)	− 0.23	< 0.001***	0.41	< 0.001***	2.91	0.004**

* N* = 284. *ρ* = Spearman correlation coefficient; *p* values are two-tailed.

*** *p* < .001, ** *p* < .01, * < 0.05. *P* values were corrected for the false discovery rate

### Psychometric properties of the GMS and AMS

*Internal consistency*: Given violations of the tau-equivalence assumption for both the GMS and the AMS, internal consistency was assessed using McDonald’s omega (), which is more appropriate for tau-congeneric measurement models than Cronbach’s alpha. For the GMS, McDonald’s omega indicated acceptable internal consistency with = 0.77. Similarly, the AMS demonstrated good internal consistency, with an omega coefficient of = 0.83.

*Split-Half Reliability*: Using the maximal split-half coefficient (λ₄) to assess split-half reliability, the highest λ₄ value (0.69) was obtained for the GMS when items 4 and 5 were grouped in one half and items 1, 2, and 3 in the other. Cross-validation in an independent sample yielded a cross-validated maximal split-half reliability of Max(λ₄) = 0.71, indicating acceptable internal consistency and improved generalizability. For the AMS, the optimal item split—grouping items 4 and 5 against items 1, 2, and 3—produced a maximal λ₄ coefficient of 0.77 in the full sample. Cross-validation yielded a higher and stable estimate of Max(λ₄) = 0.82, supporting strong split-half reliability for the AMS.

**Fig. 1:**
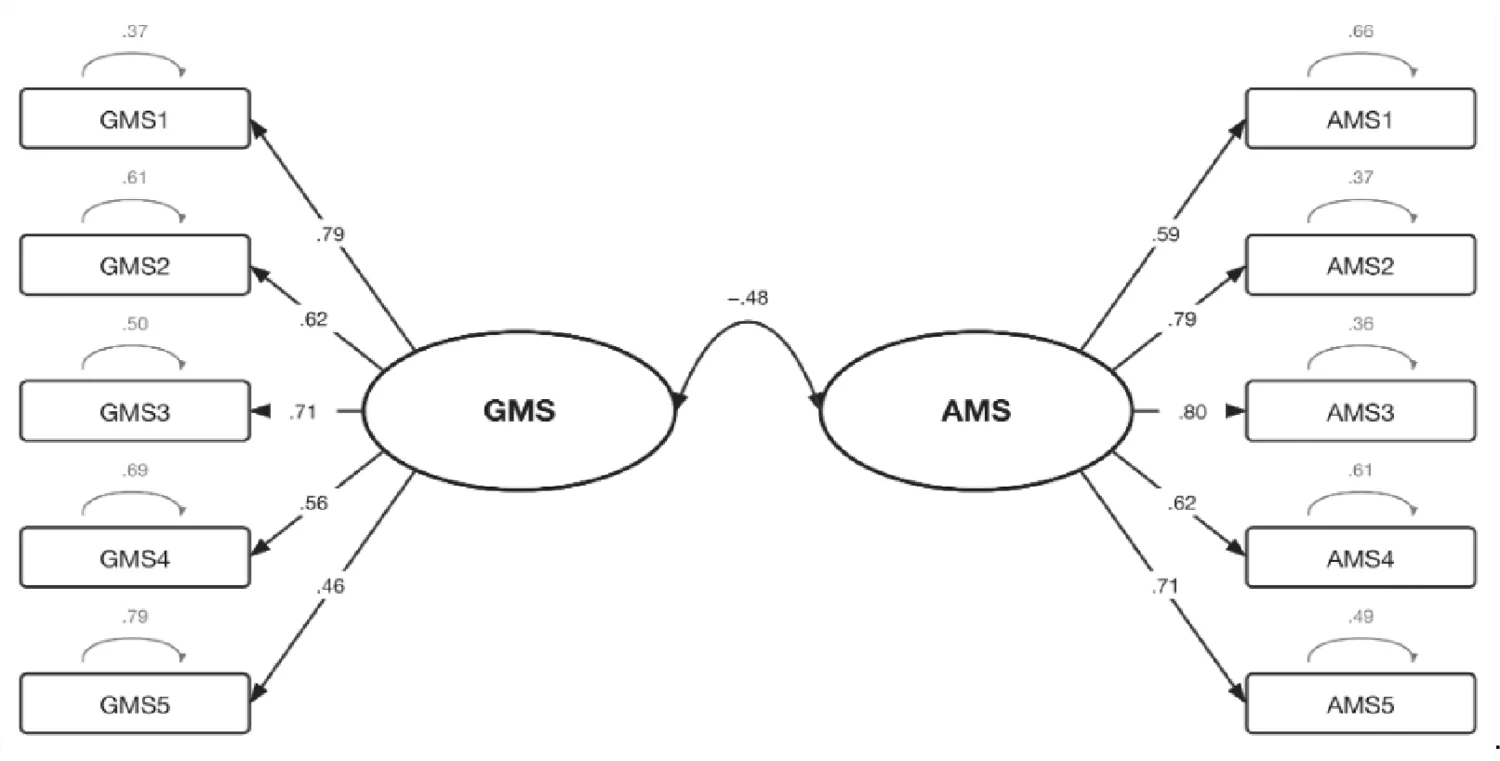
Confirmatory Factor Analysis of the General Mattering Scale (GMS) and Anti-Mattering Scale (AMS). Single-headed arrows indicate directional factor loadings (standardized). Double-headed arrow indicates covariance between latent factors. Ellipses represent latent variables. Rectangles represent observed indicator variables. Small curved arrows indicate residual variances. All loadings are standardized (β)

*Confirmatory Factor Analysis*: The one-factor model, in which all GMS and AMS items loaded onto a common latent factor, demonstrated poor model fit, χ²_(35)_ = 276.44, *p* < .001, CFI = 0.733, TLI = 0.657, RMSEA = 0.156 (90% CI 0.139 to 0.173, p-close < 0.001), SRMR = 0.113. In contrast, the two-factor model, specifying separate but correlated latent factors for mattering and anti-mattering, showed substantially improved and overall acceptable model fit, χ²_(34)_ = 77.49, *p* < .001, CFI = 0.952, TLI = 0.936, RMSEA = 0.067 (90% CI 0.047 to 0.087, p-close = 0.075), SRMR = 0.068. Comparison of the nested models indicated that the two-factor model fit the data significantly better than the one-factor model, Δχ²_(1)_ = 198.94, *p* < .001, providing evidence for the distinctiveness of mattering and anti-mattering as related but separable constructs (Fig. 1). Standardized factor loadings in the correlated two-factor model ranged from 0.46 to 0.80 (see Table 1) and were all statistically significant (*p* < .001). Inspection of standardized residuals and modification indices revealed no evidence of substantial localized model misspecification.

*Measurement invariance*: Measurement invariance results are summarized in Table 3. Across both gender and prior treatment groups, metric and scalar measurement invariance were supported, indicating comparable measurement properties of the GMS and AMS across groups.

**Table 3:** Measurement invariance for gender and prior psychiatric or psychotherapeutic treatment

Grouping	Scale	Contrast	Test statistic	*P* value
Gender	GMS/AMS	Configural	χ²_(68)_ = 108.86	
		Metric	Δχ²_(8)_ = 9.97	0.267
		Scalar	Δχ²_(8)_ = 12.35	0.136
Prior treatment		Configural	χ²_(68)_ = 123.65	
		Metric	Δχ²_(8)_ = 6.45	0.597
		Scalar	Δχ²_(8)_ = 2.98	0.936

Prior treatment includes psychiatric and psychotherapeutic treatment. Measurement invariance was evaluated using χ² likelihood-ratio tests comparing configural, metric, and scalar invariance models. Effect sizes are reported as Cohen’s d with 95% confidence intervals in parentheses

*GMS* general mattering scale, *AMS* anti-mattering scale

### Associations between child maltreatment, mattering, anti-mattering, and loneliness

Spearman rank correlation analyses revealed that GMS and AMS were significantly negatively correlated (*ρ* = –0.37), indicating that higher perceptions of mattering were associated with lower levels of perceived anti-mattering (see Table 2).

General mattering was negatively associated with loneliness (*ρ* = –0.47) and all three subscales of the DASS-21, with the strongest correlation observed for depression (*ρ* = –0.27), followed by anxiety (*ρ* = –0.24) and stress (*ρ* = –0.17). General mattering was positively associated with quality of life (*ρ* = 0.38) and the social network size (*ρ* = 0.38). A negative correlation was observed for child maltreatment (*ρ* = –0.23).

In contrast, anti-mattering was positively correlated with loneliness (*ρ* = 0.50), all DASS-21 subscales: depression (*ρ* = 0.45), anxiety (*ρ* = 0.37), stress (*ρ* = 0.34), and child maltreatment (*ρ* = 0.41), indicating that greater feelings of anti-mattering were associated with increased psychological distress and a history of child maltreatment. Anti-mattering also showed a significant negative correlation with quality of life (*ρ* = –0.49) and social network size (*ρ* = –0.21).

The comparison of correlation coefficients showed that anti-mattering showed stronger associations with psychological distress and childhood trauma than mattering (see Table 2: depressive symptoms: *r* = .45 vs. *r* = − .27; Fisher’s *z* = 2.98, *p* = .003; anxiety: *r* = .37 vs. *r* = − .24; Fisher’s *z* = 2.08, *p* = .04; stress: *r* = .34 vs. *r* = − .17; Fisher’s *z* = 2.67, *p* = .01; childhood trauma: *r* = .41 vs. *r* = − .23; Fisher’s *z* = 2.91, *p* = .004). In contrast, social network size was more strongly associated with mattering (*r* = .38 vs. *r* = − .20; Fisher’s *z* = 2.71, *p* = .007). Of note, no significant difference was found for loneliness (*r* = − .47 vs. *r* = .50; Fisher’s *z* = 0.54, *p* = .59).

### Mediation analysis

**Fig. 2:**
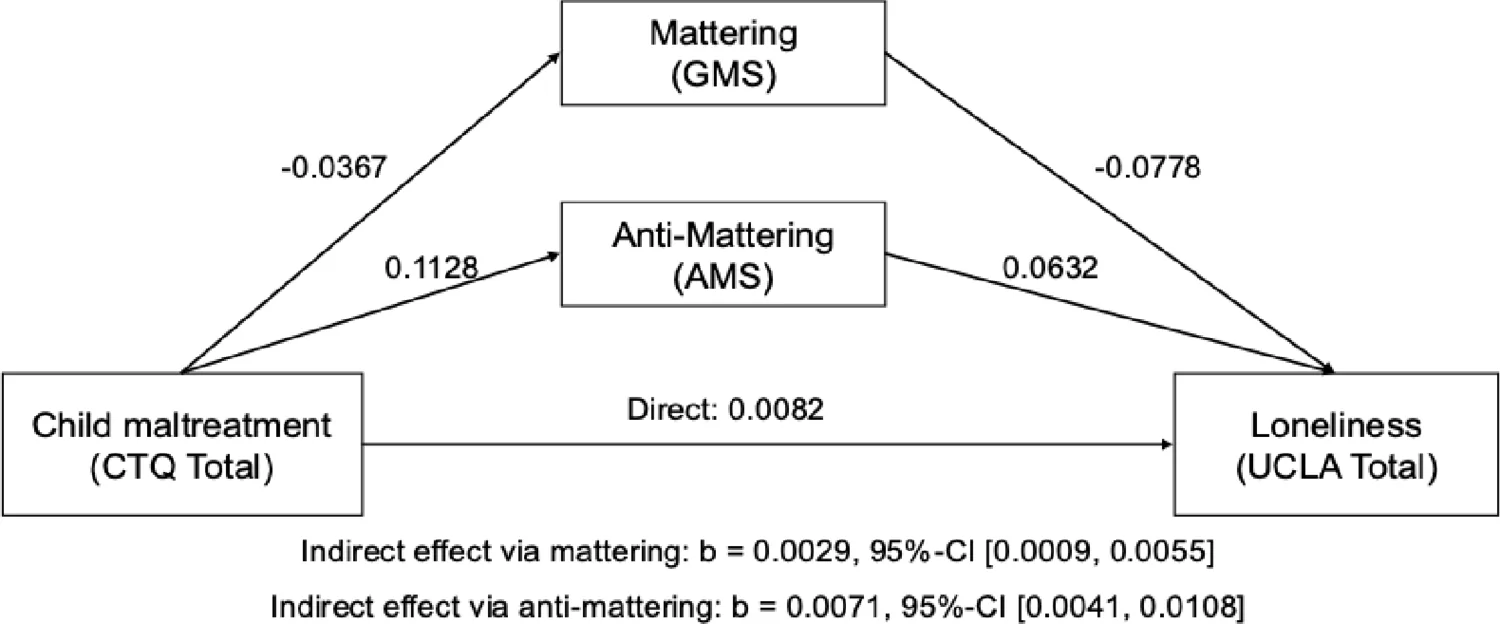
Mediation model with mattering and anti-mattering as mediators from child maltreatment to loneliness

A parallel multiple mediation analysis was conducted to analyze whether the relationship between child maltreatment and loneliness was mediated by mattering and/or anti-mattering (see Fig. 2). Regression results indicated that the total score of child maltreatment were a significant predictor of both mediators: negatively predicting mattering (*b*= − 0.037, *SE* = 0.012, *t*_[282]_ = − 3.05, *p* = .003, 95% CI [–0.060, − 0.013]) and positively predicting anti-mattering (*b* = 0.113, *SE* = 0.014, *t*_[282]_ = 7.90, *p* < .001, 95% CI [0.085, 0.141]).

The parallel multiple mediation analysis revealed that child maltreatment, mattering, and anti-mattering were all significant predictors of loneliness: child maltreatment had a direct positive effect on loneliness (*b* = 0.008, *SE* = 0.002, *t*_[280]_ = 3.38, *p* < .001, 95% CI [0.003, 0.013]). Mattering was negatively associated with loneliness (*b* = − 0.078, *SE* = 0.012, *t*_[280]_ = − 6.62, *p* < .001, 95% CI [–0.101, − 0.055]), whereas anti-mattering was positively associated with loneliness (*b* = 0.063, *SE* = 0.010, *t*_[280]_ = 6.38, *p* < .001, 95% CI [0.044, 0.083]).

Bootstrap analyses with 10,000 samples revealed significant indirect effects of child maltreatment on loneliness through both mediators. The indirect effect via mattering was *b* = 0.003 (BootSE = 0.001), with a 95% bootstrap confidence interval [0.001, 0.006], and via anti-mattering was *b* = 0.007 (BootSE = 0.002), 95% CI [0.004, 0.011]. Notably, the indirect effect through anti-mattering was more than twice the magnitude of the effect through mattering, indicating that anti-mattering was a stronger mediator in this relationship. To summarize, both mattering and anti-mattering partially mediated the relationship between child maltreatment and loneliness.

## Discussion

Our findings suggest that anti-mattering partially mediated the association between child maltreatment and loneliness and may therefore be relevant to understanding their relationship. While the German versions of the GMS and AMS demonstrated satisfactory psychometric properties, the most notable finding of the study was the stronger role of anti-mattering in explaining the association between early adversity and loneliness, supporting the notion that perceived interpersonal insignificance may represent a correlate of relational distress and psychological vulnerability.

In contrast to conceptualizations of loneliness that primarily emphasize the absence of social contact, the present findings support perspectives suggesting that loneliness is fundamentally tied to subjective experiences of interpersonal significance and emotional connectedness, as has also been proposed in previous studies [2, 3]. Individuals may experience loneliness not merely because they are socially isolated, but also because they perceive themselves as unseen, unimportant, or emotionally irrelevant to others. In this context, anti-mattering may reflect a particularly painful relational experience characterized by perceived invisibility, insignificance, and interpersonal invalidation [10].

The stronger associations observed for anti-mattering relative to mattering with depression, anxiety, and stress replicates previous findings in different countries [10–14], and may indicate that anti-mattering represents a more intensive experience that is closer to individuals’ psychological functioning [7, 10]. Whereas low mattering may primarily reflect the absence of perceived interpersonal significance, anti-mattering may involve active expectations of exclusion, neglect, and relational devaluation that could additionally be rooted in a history of child maltreatment. Persistent perceptions of interpersonal insignificance may be associated with broader difficulties involving self-worth, belongingness, and interpersonal trust.

The present findings may be particularly relevant in the context of child maltreatment. Experiences of abuse and neglect may communicate powerful relational messages that one is unimportant, invisible, or undeserving of care. Over time, such experiences may be reflected in relatively stable relational schemas [17] characterized by chronic expectations of insignificance and interpersonal disconnection. Anti-mattering may therefore be relevant to understanding the associations among adverse childhood experiences, loneliness, and broader mental health difficulties.

The findings regarding social network size further support the distinction between loneliness and objective social connections in line with previous work [37, 38]. The stronger associations of loneliness with mattering and anti-mattering underline again, that individuals may maintain social contacts and still experience profound loneliness–especially when they perceive themselves as emotionally insignificant, unseen, or disconnected from others. Subjective perceptions of interpersonal importance may be more relevant for understanding loneliness than the mere quantity of social relationships. However, a broader social network may facilitate the experience of mattering to others.

From a clinical perspective, our findings emphasize the value of assessing both the presence of mattering and the experience of anti-mattering in individuals with histories of child maltreatment and loneliness. Anti-mattering, in particular, may serve as a valuable addition to screening tools aimed at identifying individuals who feel unseen, unvalued, or socially disconnected–experiences that may possibly elevate the risk for loneliness, depressive symptoms, and broader psychological distress. Additionally, it may be useful to explore those people who have both a high level of anti-mattering and a reduced social network size as the latter may possibly reenforce the perception of being insignificant. Interventions emphasizing interpersonal validation, emotional recognition, relational connectedness, and corrective relational experiences may therefore help reduce persistent feelings of insignificance and disconnection.

In addition to the mediation findings, the German versions of the GMS and AMS demonstrated satisfactory psychometric properties, including good internal consistency and acceptable factorial validity as well as measurement invariance across gender and across participants with vs. without prior psychiatric or psychotherapeutic treatment. The additional factor analyses further supported the conceptual distinction between mattering and anti-mattering, consistent with previous research suggesting that anti-mattering reflects more than merely the absence of mattering. These findings support the suitability of the German versions for future research and clinical applications in German-speaking populations.

Several limitations should be considered. First, the cross-sectional design precludes causal inferences or assessment of temporal stability. Second, the sample lacked demographic representativeness, skewing younger and predominantly female, with a very small non-binary group, limiting generalizability to the broader German-speaking population. Third, while both scales showed good internal consistency, the split-half reliability depends on sample characteristics and should be interpreted cautiously. Finally, the use of the Social Network Index focused on the structural network size, neglecting qualitative dimensions of social relationships, which are important given the link of mattering and anti-mattering to social support.

Future studies should replicate and extend these findings using more diverse and representative samples, including varied clinical groups, age groups, cultural backgrounds and marginalized groups. People with a mental health diagnosis are particularly prone to experiencing child maltreatment and loneliness, and the presence of a feeling of anti-mattering may be associated with further distress in this vulnerable group. Longitudinal research is needed to examine the stability of mattering and anti-mattering over time and its predictive value for psychological outcomes such as depression and loneliness. Further investigation into the relationship between mattering, anti-mattering and loneliness is also warranted, given their overlapping roles in psychological distress and potential target for psychotherapeutic interventions. Incorporating (anti-)mattering assessments into therapeutic settings as part of a broader diagnostic toolkit could enhance early identification of individuals at risk and improve treatment outcomes, particularly by addressing clients’ needs for validation and belonging. Interventions focusing on affirming clients’ sense of mattering may prove effective and merit further exploration.

## Conclusion

To conclude, this study represents the first evaluation of the psychometric properties and factor structure of the GMS and AMS within a German-speaking sample. Both German scales demonstrated significant associations with loneliness, social network size, quality of life, and psychological distress. Moreover, both the GMS and especially AMS partially mediated the association of child maltreatment and loneliness. Overall, our findings highlight the potential relevance of mattering-related constructs in understanding the long-term psychosocial consequences of adverse childhood experiences.

## Data Availability

The datasets generated and/or analyzed during the current study are available from the corresponding author on reasonable request.
